# Patriotic

**Published:** 1885-07

**Authors:** 


### Patriotic.

In view of the deep interest manifested throughout the country, respecting the completion of the pedestal for *the Statue of Liberty*, the committee having the matter in charge have decided to issue a perfect model of the statue and its pedestal, which they can fully commend to all lovers of liberty and art.

Orders for this model can be obtained by addressing Mr. Richard Butler, Secretary of the Committee, No. 55 Liberty street, New York.

